# Individual Differences in Auditory Training Benefits for Hearing Aid Users

**DOI:** 10.3390/clinpract13050107

**Published:** 2023-09-29

**Authors:** Ayelet Barda, Yair Shapira, Leah Fostick

**Affiliations:** 1 Department of Health Management, Ariel University, Ariel 40700, Israel; ayelet.naveh@msmail.ariel.ac.il (A.B.); yairsh@ariel.ac.il (Y.S.); 2 Department of Communication Disorders, Auditory Perception Lab in the Name of Laurent Levy, Ariel University, Ariel 40700, Israel

**Keywords:** individual differences, auditory training, speech perception, generalization

## Abstract

The present study aimed to examine whether individual differences in baseline speech perception could serve as predictors for the effectiveness and generalization of auditory training (AT) to non-trained tasks. Twelve adults, aged 60–75 years with bilateral hearing loss, completed a two-month, home-based, computerized AT program, involving sessions four times per week. Training tasks included the identification of vowel frontal, height, manner of articulation, point of articulation, voicing, and open-set consonant-vowel-consonant (CVC) words. Non-trained speech perception tests were conducted one month before AT, prior to training, after one and two months of training, and during a two-month follow-up. The results showed that one month of AT improved performance in most trained tasks, with generalization observed in the CVC words test and HeBio sentences with speech-shaped noise (SSN). No evidence of spontaneous learning or added benefit from an extra month of training was found. Most importantly, baseline speech perception predicted improvements in both training and post-training generalization tasks. This emphasizes the significance of adopting an individualized approach when determining the potential effectiveness of AT, applicable in both clinical and research contexts.

## 1. Introduction

The main difficulty reported by hearing aid (HA) users is speech perception, especially in auditorily challenging environments. This difficulty often affects participation in social, leisure, and occupational activities [1,2,3,4] and cannot be explained by hearing level alone [1,4,5]. Individual characteristics such as age, and the onset, duration, severity, and etiology of hearing loss, as well as one’s experience with the HA device and cognitive decline [6,7,8,9], affect speech perception [10,11,12,13]. Auditory training (AT) can improve speech recognition in people with hearing loss who use HA or implants [13]. Both new and experienced HA users enjoy AT [14,15], although a larger effect has been observed among new users [14]. AT varies regarding the auditory stimuli used, including non-linguistic sounds and stimuli related to speech [16,17]. An analytic AT includes sub-lexical features of speech (bottom-up abilities) [18], and synthetic AT involves the recognition and understanding of words, sentences, paragraphs, or conversations (top-down abilities). AT also varies in regard to frequency and duration [18], ranging from less than ten training sessions [19,20,21] to more than forty [22,23].

A critical factor in any training process is generalization, that is, the translation of the training into a difference in general daily functioning. The challenge with most previous AT studies is that learning was specific to the trained tasks, and generalization to untrained tasks was limited [24,25,26,27]. This casts doubt on the effectiveness of AT [27]. However, the individual characteristics mentioned above [6,7,8,9,10,11,12,13], as well as one’s experience with the HA device and cognitive decline, affect speech perception [28,29]. It can be argued that the null finding in generalization may be due to the inclusion of a large range of individual differences among participants that can affect hearing ability, speech perception, and training benefits. Therefore, the current study aims to focus on the effect of individual differences when testing the benefits of AT. The novelty of this study is testing whether individuals’ baseline speech perception is related to improvements in AT and the degree of its generalization to non-trained speech perception tasks.

## 2. Materials and Methods

### 2.1. Participants

Twelve adults (eight women, four men) with mild to profound post-linguistic hearing loss who used two HA were enrolled in this experiment (Table 1). This sample size has a power of 0.7, based on the average effect sizes obtained in the study. Nine participants had sensorineural hearing loss, and three had mixed hearing loss. Their age range was 60–75 years (M = 68.42, SD = 4.76), and they were all native Hebrew speakers or had spoken Hebrew for over twenty years. All participants were able to use a computer and did not present significant cognitive impairment (Montreal cognitive assessment [Moca] test score greater than 26; M = 28.17, SD = 1.03) [30]. Participants were recruited through social media and advertising in audiology clinics and were from diverse residential areas.

### 2.2. Training

The training was conducted using six tasks: (1) identification of vowel frontal; (2) identification of vowel height; (3) identification of manner of articulation; (4) identification of point of articulation; and (5) identification of voicing. In all these tasks, participants chose the right answer from a closed set presented on the screen. The sixth task, involving an open set, was the (6) identification of open set CVC words, in which participants were asked to type the uttered CVC word on a computer keyboard. To avoid errors related to typos, participants could correct their answers and then approve and submit them. Each task had 10 items selected randomly out of 50–60 items at each training session. All items were auditory-only (no visual information was available) and were of a similar difficulty level. The tasks were performed using the HEARO™ website, which provides online auditory training for Hebrew-speaking adults (Hearo.co.il). The stimuli in these six training tests were presented amidst two-talker babble noise (2TBN). Participants received training for all six tasks in each training session.

To keep a naturalistic setting that could be generalized to clinical use, the participants conducted the AT on their personal computers, used hearing aids in the regular program, and listened to the stimuli through the computer’s loudspeaker. Prior to study initiation, the researchers checked that the HA was working properly. The participants were instructed to perform the AT in a quiet place at their home, four days per week over eight weeks, without specific instructions regarding the time of day and day of the week. Each training session lasted about 45 min. A research assistant contacted the participants during the week, at their convenience, to ensure they performed the training. A feedback score for each task was given at the end of each training session and was saved on the website. All participants completed all training sessions. During the first AT, the training stimuli (the words) were calibrated to a comfortable level (about 20 dB above the speech reception threshold, minimum 65 dB SPL), and the 2TBN were calibrated to an intensity that would provide 50% accuracy in each training task, demonstrating the participant’s speech reception threshold in noise (SRTn).

### 2.3. Assessment of Speech Perception (Generalization)

Speech perception (generalization) was assessed using the HeBio sentences test, CVC words in quiet test, and the digits in noise (DIN) test.

The HeBio sentence test. The Hebrew adaptation of the AzBio test of understanding (English) sentences in noise conditions [31,32]. The test includes 33 sentence lists of 3–12 words (Mean = 7.0, SD = 1.4). Each list includes twenty sentences, five from each of the four talkers (two men, two women). The participants are asked to repeat the sentence they heard and to guess when they did not hear well. In each testing session, two lists of sentences were presented, one amidst speech-shaped noise (SSN) and one amidst four-talker babble noise (4TBN). SRTn was measured using an adaptive procedure of presenting the speech stimulus with noise. The initial SNR was 10 dB, and the SRTn (corresponding to 50% correct responses) was the average SNR of the last ten sentences of the twenty presented [30].

CVC words in quiet. This test consists of twenty lists of 10 meaningful, one-syllable, consonant–vowel–consonant phonetically balanced Hebrew words, constructed according to the Arthur Boothroyd words test [33] (i.e., in each list, every consonant appears once, and every vowel appears twice). This test was different from the CVC words training tasks, containing different words presented without background noise (unlike the training performed at 2TBN). For each participant, two lists of 10 words were presented. Participants were instructed to repeat each word they heard and guess if they were unsure. The results were manually recorded by the experimenter (first author) and expressed as the percentage of correct words.

DIN is a speech-in-noise test that uses digit triplets in steady-state speech noise [34]. In the current study, we used the Hebrew version of the test [35]. The test measures the speech reception threshold in long-term average speech-spectrum noise (SRTn) via a 1-up, 1-down adaptive procedure with a measurement error of 0.7 dB [35]. The stimuli level was set at 65 dB SPL. It was first delivered in two practice trials with a fixed SNR of +10, then two additional practice trials with steps of 4 dB. In the test, the initial SNR was 0 dB, with steps of 2 dB. SRTn was defined as the signal-to-noise ratio (SNR) between speech and noise display levels, where 50% of the answers were correct, with lower SNR indicating better performance.

The experimenter (first author) conducted the speech perception (generalization) tasks in an inner, quiet room in the participant’s home. The stimuli were presented at 65 dB SPL through a loudspeaker positioned at 0 azimuths and a 1 m distance from the participants while they used their personal hearing aids in the regular program. The tests lasted 45 min. The set-up was the same between the participants, except for the order of the tests, which was random. Different sentence and word lists were presented in the HeBio sentences and CVC word tests in each test session.

### 2.4. Overall Design

**Figure 1:**
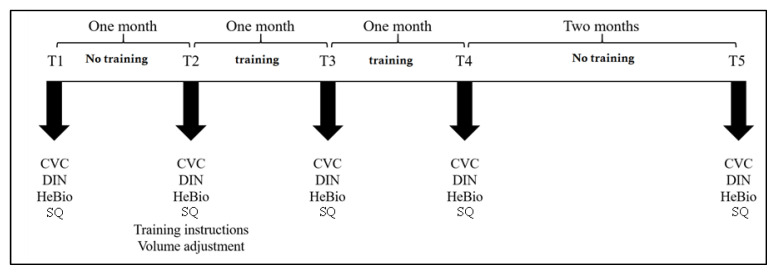
Illustration of the study procedure. T = test session; CVC = consonant–vowel–consonant words; DIN = digit-in-noise test; HeBio sentences test (Reprinted/adapted with permission from Ref. [36]).

The overall design of the current study follows that reported in [36]. Participants provided signed informed consent to participate in the study, answered a personal details questionnaire, and presented a hearing audiogram performed within the last six months to verify their hearing threshold and suitability for participation in the study. Upon qualifying, participants completed the AT four times per week over eight weeks. To control for procedural learning, pre-training baseline performance was measured twice, one month apart, before participants started the training program (i.e., T1 and T2). The training effect was reassessed after one month of AT (T3) and again after the completion of a second month of AT (T4). Training retention was assessed after two months of no training (T5) (Figure 1). Participants received a free bi-monthly subscription to the AT website and a small monetary compensation for their participation in the study.

### 2.5. Data Analysis

The Kolmogorov–Smirnov test for normality revealed that, when tested against α = 0.05, 61% of the training tests (comprising six tests across 32 sessions) exhibited distributions that were not significantly different from normal. When correcting for multiple comparisons to control the Type I error rate, this percentage increased to 91%. A similar pattern was observed for the speech-perception (generalization) tasks, with 90% of their distributions not differing significantly from normal at α = 0.05 and 91% when adjusting for multiple comparisons. Therefore, analysis of variance was applied for all group comparisons, and Pearson correlations were applied for individual analyses.

## 3. Results

### 3.1. Group Analysis

**Figure 2:**
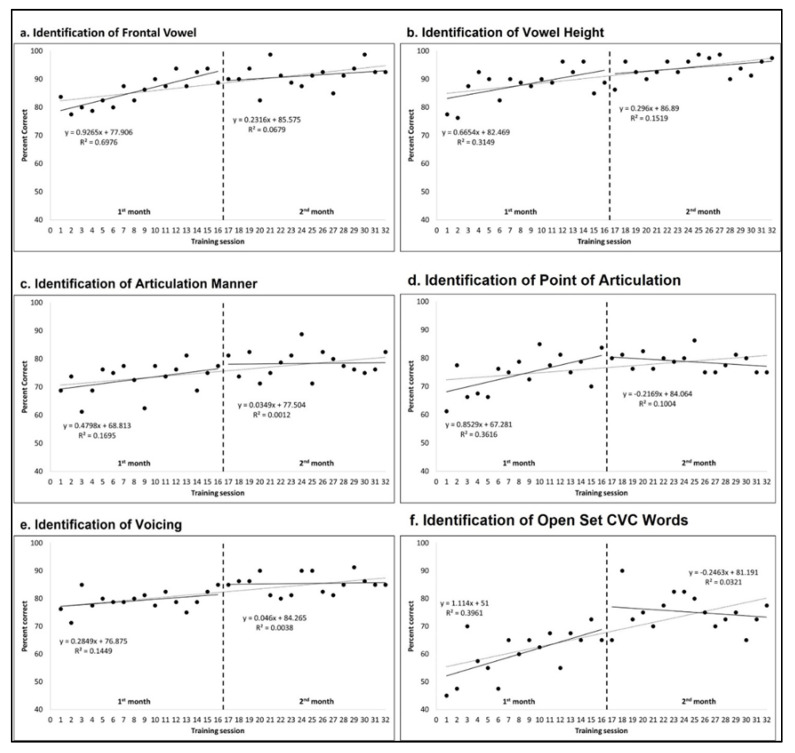
Accuracy scores and statistical analyses across all training sessions for the six training tasks: (**a**) identification of frontal vowel; (**b**) identification of vowel height; (**c**) identification of articulation manner; (**d**) identification of point of articulation; (**e**) identification of voicing; and (**f**) identification of open set CVC words.

To test the improvement in training tasks, a two-way repeated measures MANOVA was performed across all 32 training sessions and separately for the first and second months of training, with accuracy scores on the six training tasks as dependent variables (Figure 2). A significant main effect of training was found across all 32 weeks (F(186,1302) = 1.573, *p* < 0.001, partial ɳ^2^ = 0.183). This improvement was due to an improvement in the first month of training, where there were improvements shown in all tasks (F(90,900) = 1.615, *p* < 0.001, partial ɳ^2^ = 0.139). No improvement was observed in the second month of training (F(90, 630) = 1.122, *p* = 0.221, partial ɳ^2^ = 0.138).

A two-way repeated measures univariate ANOVA for each task, carried out during the first month of training, showed increased accuracy scores across training sessions for Identification of Vowel Frontal (F(15,50) = 2.064, *p* = 0.015, partial ɳ^2^ = 0.171), Identification of Vowel Height (F(15,50) = 2.169, *p* = 0.010, partial ɳ^2^ = 0.178), Identification of Point of Articulation (F(15,50) = 3.077, *p* < 0.001, partial ɳ^2^ = 0.235), Identification of Open Set CVC Words (F(15,50) = 2.199, *p* = 0.009, partial ɳ^2^ = 0.180) but not for the Identification of Manner of Articulation (F(15,50) = 1.050, *p* = 0.408, partial ɳ^2^ = 0.095) and Identification of Voicing tasks (F(15,50) = 1.182, *p* = 0.292, partial ɳ^2^ = 0.106).

**Figure 3:**
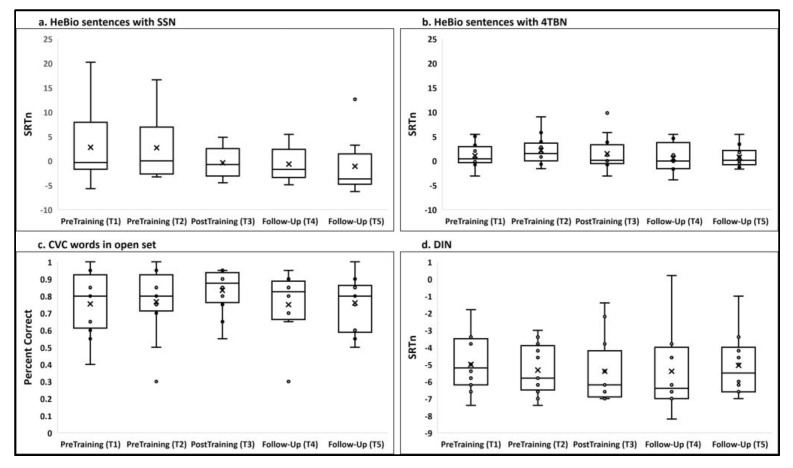
The mean, median, and interquartile range in each testing session of (**a**) HeBio sentences with SSN (SRTn); (**b**) HeBio sentences with 4TBN (SRTn); (**c**) CVC words in quiet (accuracy); (**d**) DIN (SRTn). Participants’ individual scores are represented by circles; the mean is presented by the x-sign.

Figure 3 presents box plots of the four speech-perception (generalization) tasks in the five testing sessions. A two-way repeated measures MANOVA showed that a significant general main effect was found for the session (F(16, 112) = 1.791, *p* = 0.041, partial ɳ^2^ = 0.204). However, the session effect was significant only for CVC words (F(4,28) = 3.107, *p* = 0.050, partial ɳ^2^ = 0.307) and not for HeBio sentences with SSN (F(4,28) = 2.776, *p* = 0.113, partial ɳ^2^ = 0.284) and with 4TBN (F(4,28) = 2.531, *p* = 0.137, partial ɳ^2^ = 0.266), and for DIN (F(4,28) = 0.410, *p* = 0.694, partial ɳ^2^ = 0.055).

### 3.2. Individual Analysis

**Figure 4:**
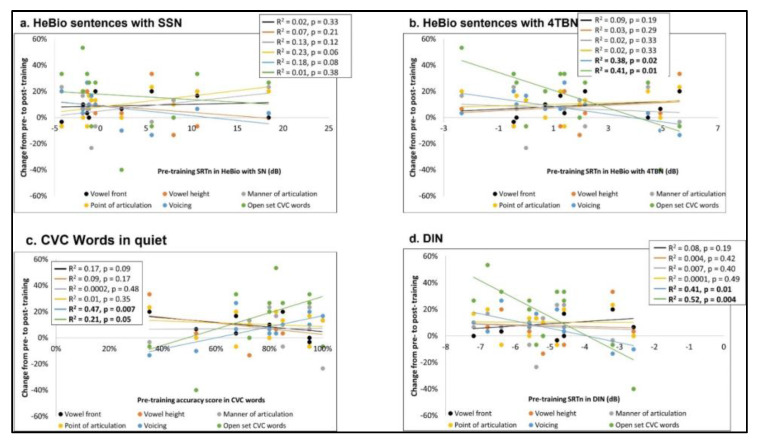
The change in the six training tasks according to participants’ baseline scores in the speech perception (generalization) tasks: (**a**) HeBio sentences with SSN (SRTn); (**b**) HeBio sentences with 4TBN (SRTn); (**c**) CVC words in quiet (accuracy); (**d**) DIN (SRTn). Bold regression analysis results indicate a significant relationship.

**Figure 5:**
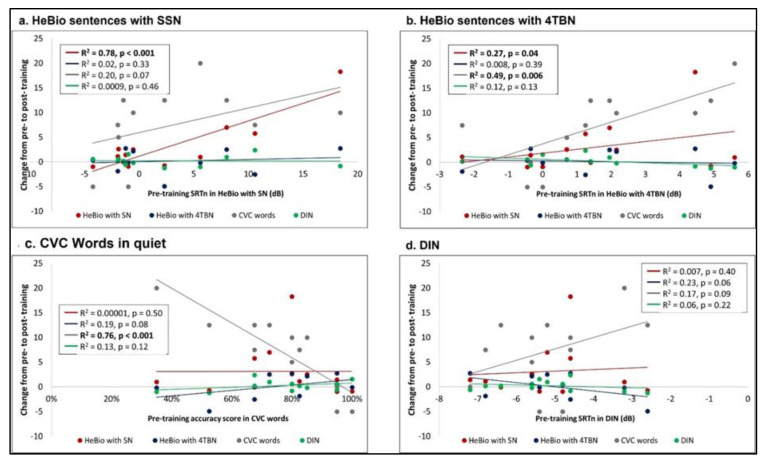
The change in the speech perception (generalization) tasks according to participants’ baseline scores in the speech perception (generalization) tasks: (**a**) HeBio sentences with SSN (SRTn); (**b**) HeBio sentences with 4TBN (SRTn); (**c**) CVC words in quiet (accuracy); (**d**) DIN (SRTn). Bold regression analysis results indicate a significant relationship.

Since no difference was found between T1 and T2 and between T3 and T4 in the generalization tasks, they were averaged, creating pre-training and post-training scores. Correlation analyses between the pre-training scores of the generalization tasks and the change (post-training score—pre-training score) in the training tasks (Figure 4) and in the generalization tasks (Figure 5) are summarized in Table 2. The results showed that CVC word accuracy and DIN predicted improvements in the voicing and open set CVC words training tasks, showing larger improvements when the baseline speech perception (generalization) was better (lower DIN SRTn and higher CVC words accuracy). No such relationships were observed between the pre-training score of the HeBio sentences test with SSN or 4TBN and training tasks (Figure 4).

Correlation analyses between the pre-training scores of the speech perception (generalization) tasks and the change that occurred in them during training (post-training score—pre-training score) revealed an opposite relationship to that found in the training tasks: a lower pre-training score predicted larger changes in the speech perception (generalization) tasks. Pre-training HeBio sentences with SSN predicted improvement in HeBio sentences with 4TBN; pre-training HeBio sentences with 4TBN predicted improvement in HeBio sentences with SSN; pre-training CVC words predicted improvement in CVC words (Figure 5).

## 4. Discussion

Most AT studies fail to show the generalization of the training to untrained stimuli. The present study was the first to test whether individual differences in baseline speech perception are related to the benefits of AT. The results showed that the baseline speech perception of older adults using HA was related to their improvement in the AT, and, more importantly, to their improvement in the non-trained speech perception (generalization) tasks. Group analyses showed improvements in the AT but a limited generalization effect. They also showed that one month of training was enough, with no additional effects seen for the second month of training.

Generalization is the biggest challenge of AT, and the scientific literature reflects mixed evidence regarding the ability to successfully generalize AT to untrained speech perception (generalization) tasks [25,27,29,37,38,39]. The underlying assumption of the present study was that the low generalization of AT is due to large individual differences in the trained population. Indeed, when participants in the present study were tested as a group, as in previous studies, generalization was observed only to the perception of CVC words presented with no background noise, which was very similar to the trained stimuli but not to the other generalization tasks. However, when the analysis considered the participants’ individual level in the pre-training speech perception, it showed that lower baseline speech perception predicted larger generalization, demonstrated by a significant improvement in the non-trained speech perception (generalization) tasks, albeit a lower improvement in the training tasks. It is possible that the training tasks were too difficult for participants with lower baseline speech perception, making it difficult for them to show any improvement. Nevertheless, the training influenced these participants and improved their auditory abilities, as was reflected in the generalization tasks. This can be explained by the reverse hierarchy theory [40,41], suggesting that difficult tasks during training make learning more specific to trained features in perceptual learning, and easier tasks during training allow generalization to occur [41].

Similar to previous studies, improvements in AT were observed following one month of training [26,39,42,43,44]. The present study also showed that an additional month did not add to this improvement, suggesting that one month is sufficient for AT. Therefore, more extended training has minimal or no benefit. Spontaneous learning was not evident in any task, suggesting that the improvement observed later was due to training. A lack of spontaneous learning in speech perception was reported earlier by some researchers [45] but not by others [26,45]. Also, similar to previous studies, an improvement in the training tasks was observed [27]. The improvement was not observed in tasks requiring the identification of manner of articulation and voicing. This might be due to the consonants included in these tasks, mostly with high frequencies (/s/,/ts/,/x/,/v/,/f/,/z/). High-frequency consonants are difficult to perceive with HA, so they are likely to be insensitive to training; this is supported by previous findings showing that older HA users improved regarding their phoneme discrimination in noise for all phonemes except those with high frequencies [25].

It should also be noted that the improvement in the training tasks was not linear and consistent, albeit there was general improvement throughout the month. Such within-training variance has been observed previously [26,46] and can be due to changes in attentional and motivational factors and the timing of training. Also, auditory temporal perception has been found to be sensitive to a diurnal rhythm, as reflected in performance changes during different hours of the day [47]. In the present study, participants were not instructed to conduct their training at a specific time of day. This was decided in order to maintain the naturalistic nature of the study and its generalization to clinical settings. However, it may have affected the training benefit. Future training studies should consider controlling the timing of the training hours to more effectively assess this factor’s possible influence. Other limitations of the study include its relatively small number of participants and naturalistic nature. The participants who volunteered for the study had to agree to two months of daily training. This resulted in a relatively small number of participants overall. Although this sample size is similar to previous studies in the field [18,26], it may have obscured additional effects. Thus, a larger group of subjects is required to confirm the conclusions obtained in the current study. In addition, since participants were recruited on a volunteering basis, and not from a certain institution, information about their hearing aid fitting was lacking. Future studies should also consider the resonance of the external auditory canal [48], the fitting of the hearing aid [49], and the use of extended-wear hearing aids.

## 5. Conclusions

Individual differences are common and can affect post-training performance at the group level. Therefore, an individual approach should be taken regarding post-training and generalization testing. A one-month training period seems sufficient for AT, and more extensive training does not appear necessary. In addition, the limitation of HA at high frequencies can affect training gains. There is little point in training participants with sounds they cannot hear. Therefore, the frequency of speech sounds should be considered when designing training protocols. These findings suggest that most of the improvement resulting from the AT was in the trained tasks. Accordingly, it is recommended that the training tasks chosen are similar to what is required of the person in terms of everyday auditory function. Most importantly, it is recommended to consider the listener’s basic speech perception ability when choosing their auditory training, and to adjust preliminary expectations appropriately based on the patient.

## Figures and Tables

**Table 1:** Demographics of the research sample.

ID	Age (Years)	Gender	Year Since First Diagnosis	Etiology of Hearing Loss	Hearing Loss Level	
					Right	Left
1	68	F	7	Hole in the eardrum	Mild to moderate	Mild to moderate
2	66	M	4	Unknown	Mild	Mild to moderate-severe
3	65	F	15	Unknown	Moderate to moderate-severe	Mild to moderate-severe
4	70	M	30	Calcification	Moderate-severe to profound	Mild to severe
5	74	F	11	Accumulation of fluid in the brain	Mild to moderate	Moderate to moderate-severe
6	72	F	12	Unknown	Moderate-severe	Moderate to moderate-severe
7	60	F	10	Genetic	Moderate	Moderate
8	70	M	46	Unknown	Mixed, Minimal to severe	Minimal to severe
9	68	M	47	Noise exposure	Minimal to profound	Minimal to severe
10	61	F	8	Chemotherapy	Mild to severe	Moderate-severe
11	72	F	4	Unknown	Mild to moderate	Mild to moderate
12	75	F	5	Unknown	Moderate	Minimal to mild
Mean	68.42	M: 33.3% F: 66.6%	16.58			
SD	4.76		15.62			

**Table 2:** Summary of R^2^ (p) for the individual analysis: change in identification of vowel frontal, identification of vowel height, identification of manner of articulation, identification of point of articulation, identification of voicing, identification of open set CVC words, HeBio with SSN, HeBio with 4TBN, CVC words, and DIN by pre-training scores of HeBio with SSN, HeBio with 4TBN, CVC words, and DIN.

	Pre-Training Scores of:			
	HeBio with SSN	HeBio with 4TBN	CVC Words	DIN
Change in:				
Identification of Vowel Frontal	0.02 (0.33)	0.09 (0.19)	0.17 (0.09)	0.08 (0.19)
Identification of Vowel Height	0.07 (0.21)	0.03 (0.29)	0.09 (0.17)	0.004 (0.42)
Identification of Manner of Articulation	0.13 (0.12)	0.02 (0.33)	0.0002 (0.48)	0.007 (0.40)
Identification of Point of Articulation	0.23 (0.06)	0.02 (0.33)	0.01 (0.35)	0.0001 (0.49)
Identification of Voicing	0.18 (0.08)	0.38 (0.02)	0.47 (.007)	0.41 (0.01)
Identification of Open Set CVC Words	0.01 (0.38)	0.41 (0.01)	0.21 (0.05)	0.52 (.004)
HeBio with SSN	0.78 (.001)	0.27 (0.04)	0.00001 (0.50)	0.007 (0.40)
HeBio with 4TBN	0.02 (0.33)	0.008 (0.39)	0.19 (0.08)	0.23 (0.06)
CVC words	0.20 (0.07)	0.49 (.006)	0.76 (.001)	0.17 (0.09)
DIN	0.0009 (0.46)	0.12 (0.13)	0.13 (0.12)	0.06 (0.22)

## Data Availability

Data from the study can be shared with interested parties following personal communication with the corresponding author.
